# Assessing the feasibility of CRISPRa approaches to enhance protein-based biomaterial expression in bacterial systems for more efficient production

**DOI:** 10.1016/j.mtbio.2025.101720

**Published:** 2025-03-29

**Authors:** Pablo Rodríguez-Alonso, Viktoriya Chaskovska, Desiré Venegas-Bustos, Alba Herraiz, Matilde Alonso, Jose Carlos Rodríguez-Cabello

**Affiliations:** a Bioforge Lab (Group for Advanced Materials and Nanobiotechnology), Laboratory for Disruptive Interdisciplinary Science (LaDIS), CIBER‐BBN, Edificio LUCIA, Universidad de Valladolid, Valladolid, 47011, Spain; b Technical Proteins Nanobiotechnology S.L., Valladolid, Spain

## Abstract

Recombinant protein production is crucial for biomedical and industrial applications; however, achieving high yields for complex protein-like biomaterials such as elastin-like recombinamers (ELRs) remains challenging. ELRs, protein-based polymers derived from tropoelastin, emulate the mechanical and bioactive properties of natural tissues, making them valuable for numerous uses. Despite their promise, implementing a sophisticated molecular system for ELR production in *Escherichia coli* involves overcoming multiple hurdles, including metabolic bottlenecks and low yields. In this study, we employed a CRISPR activation (CRISPRa) system to enhance ELR expression in *E. coli*. Although further optimization is required to reach industrial-scale outputs, our findings establish a proof of concept for taking advantage of CRISPRa to boost recombinamers yields. Such improvements represent a crucial step toward scalable production, facilitating the commercial adoption of ELRs and, in general, recombinamers not only in biomedical applications but also in broader industries that stand to benefit from these versatile biomaterials.

## Graphical abstract

**Figure:**
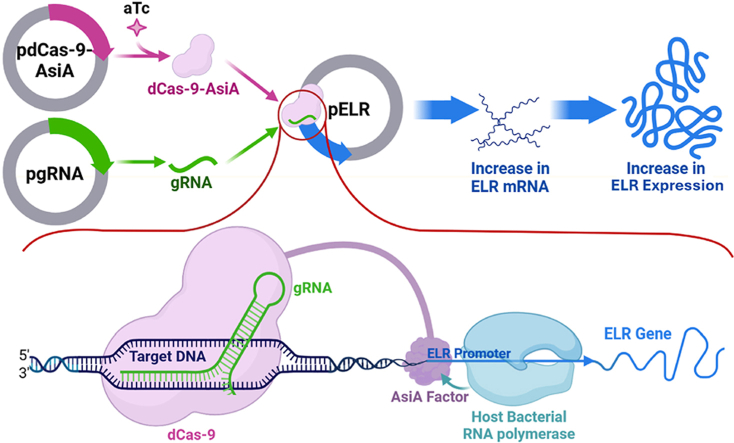

## 1 Introduction

Recombinant protein production serves as a fundamental tool for the discovery, development, and large-scale manufacture of drugs, vaccines, and research resources. Bacterial systems, particularly *Escherichia coli*, have been commonly employed to produce recombinant proteins [1]. This platform has been extensively used in the pharmaceutical industry for drug manufacturing, with recombinant human insulin, approved in 1982, serving as the first product of its kind and setting a significant precedent for heterologous protein production [2].

In this context, the growing demand for advanced biomaterials in biomedical, pharmaceutical, and industrial applications has made the development of efficient and scalable production systems a critical priority [3,4]. One of the many types of proteins that have been successfully produced are the polypeptide-based biomaterials known as elastin-like recombinamers (ELRs) [5]. These recombinantly expressed protein polymers (“recombinamers”) are composed of repeated amino acid sequences found in the intrinsically disordered regions of tropoelastin (a natural extracellular matrix component). They often include multifunctional domains that confer additional properties such as specific bioactivity, stimulus responsiveness, self-assembly capacity, or mechanical characteristics designed to mimic those of various tissues. The most representative of these “amino acid blocks” is the pentapeptide (VPGVG) (or its permutations). A wide variety of recombinamers have been developed and described with a core composition based on the general formula (VPGXG)n, where X represents any natural amino acid except proline [6].

In these biomaterials, many of their key features derive from the properties of the natural protein. For instance, the cross-linked matrices of these polymers retain many of elastin's remarkable mechanical characteristics [7], which become particularly relevant when combined with other desirable attributes such as biocompatibility [8], stimuli-responsive behavior, and the ability to self-assemble into diverse nano- and microstructures [6] and have demonstrated excellent performance in recent years, outclassing more traditional biomaterials documented, for example, by Contessotto et al. 2021 [9]. Although these proteins have been successfully produced using *E. coli*, ELR production still faces notable challenges, including cellular toxicity, and inefficient yields during heterologous expression in bacterial systems [10]. Furthermore, any increase in yield directly impacts manufacturing costs by reducing them, thereby enhancing the translational potential of these materials. Lower production costs could also expand the applications of these highly functional biomaterials beyond the biomedical sector.

To achieve heterologous expression in bacterial systems, the gene sequence encoding the desired protein must be cloned into an appropriate vector that ensures correct and efficient expression. These expression vectors are engineered to include several key features, such as a promoter, an origin of replication, a selection marker, and prokaryotic regulatory sequences, which collectively optimize recombinant protein production [1].

Among various expression systems in *E. coli*, one of the most widely used is the T7 RNA polymerase (T7) system. Although the T7 expression system is highly efficient and specific, it has some limitations compared to native bacterial expression. For instance, the high specificity and rapid transcription rate of T7 RNA polymerase can lead to cellular toxicity, plasmid instability [11,12], and the formation of inclusion bodies [13] which could present problems in some cases. Furthermore, the requirement for introducing T7 polymerase into the host increases system complexity and burdens the cellular machinery [14].

By contrast, the native *E. coli* expression system, though less efficient and yielding lower protein levels, can be more suitable for large-scale industrial applications [15,16]. It is generally more compatible with the host's physiology, enables a more moderate control of expression, and helps mitigate issues related to toxicity.

The CRISPR (clustered regularly interspaced short palindromic repeats)-Cas (CRISPR-associated) nucleases system is a groundbreaking tool for genetic manipulation, originally discovered in bacteria as a defense mechanism against bacteriophages [17]. The best-characterized CRISPR-Cas system is CRISPR-Cas9 from Streptococcus pyogenes (SpCas9) [18]. This system functions as an endonuclease that cleaves DNA at specific sites guided by a single guide RNA (sgRNA), which contains two functional regions: one that recognizes the DNA target via Watson-Crick base pairing and another that binds the Cas9 protein [19].

Alternative CRISPR systems have been engineered to modulate gene expression without inducing double-strand breaks, thus expanding the versatility of the technology. One such system is CRISPR activation (CRISPRa), designed specifically to enhance gene expression. CRISPRa uses a catalytically inactive form of Cas9, known as dead Cas9 (dCas9), which still binds specific DNA sequences via a guide RNA (gRNA) but lacks nuclease activity. Acting as a DNA-binding scaffold, dCas9 is fused to transcriptional activators to upregulate gene expression. By targeting gene promoters, CRISPRa substantially increases transcription, making it a powerful tool for investigating gene regulation and manipulating genetic pathways [20].

Although CRISPR-Cas-mediated gene activation has been extensively studied in eukaryotes, its application in prokaryotes remains comparatively underdeveloped. Reported CRISPRa systems in prokaryotic organisms typically employ dCas9 as a scaffold to target specific promoter regions via a gRNA and use various transcriptional activators to boost gene expression. For example, the RNA polymerase ω subunit (RpoZ) has been employed to target sigma70-dependent promoters [21], while bacterial enhancer-binding proteins (bEBPs) have been engineered to activate sigma54-dependent promoters [22]. Another approach leverages the SoxS transcription activator, in which a specialized scRNA not only directs dCas9 to the target DNA via its gRNA-like component but also incorporates an MS2 domain that recruits SoxS through an MCP-SoxS fusion protein [21].

Among the different CRISPRa strategies in prokaryotes, particular attention has focused on those involving the transcriptional activator AsiA, which can significantly boost basal gene expression—up to 200-fold—by binding to RNA polymerase (RNAP) [23]. By genetically fusing dCas9 with AsiA, the CRISPRa system directs dCas9–AsiA to target DNA through a gRNA, enabling AsiA to facilitate assembly of the transcriptional complex at the transcription start site (TSS) in the promoter region. In these experiments, the performance of the CRISPRa system and promoter output have been validated with GFP, β-galactosidase, and ethanol as reporters, thereby confirming the functionality of CRISPRa in prokaryotes [[21], [22], [23], [24]].

Building on these findings, our goal was to explore whether CRISPRa can feasibly enhance the native bacterial expression system for producing elastin-like recombinamers (ELRs). Although CRISPRa has shown promise in various contexts, applying it specifically to ELR production represents a non-trivial challenge. In particular, this approach requires the introduction of three separate plasmids—one encoding dCas9–AsiA, one encoding the gRNA, and one encoding the actual ELR gene—each with its own origin of replication and selection marker. Balancing these plasmids for stable maintenance and efficient expression poses a significant hurdle. Nonetheless, if successful, this strategy could provide a pathway to further optimize ELR (or any other recombinamer) yields and reduce production costs. Accordingly, our proof-of-concept study investigates the feasibility of creating a functional CRISPRa-based expression platform in *E. coli* for an elastin-like recombinamer, highlighting both the potential benefits and the complexities of this multi-plasmid system.

## 2 Results

### 2.1 CRISPRa system design as a platform for enhancing ELRs production in *E. coli*

**Fig. 1:**
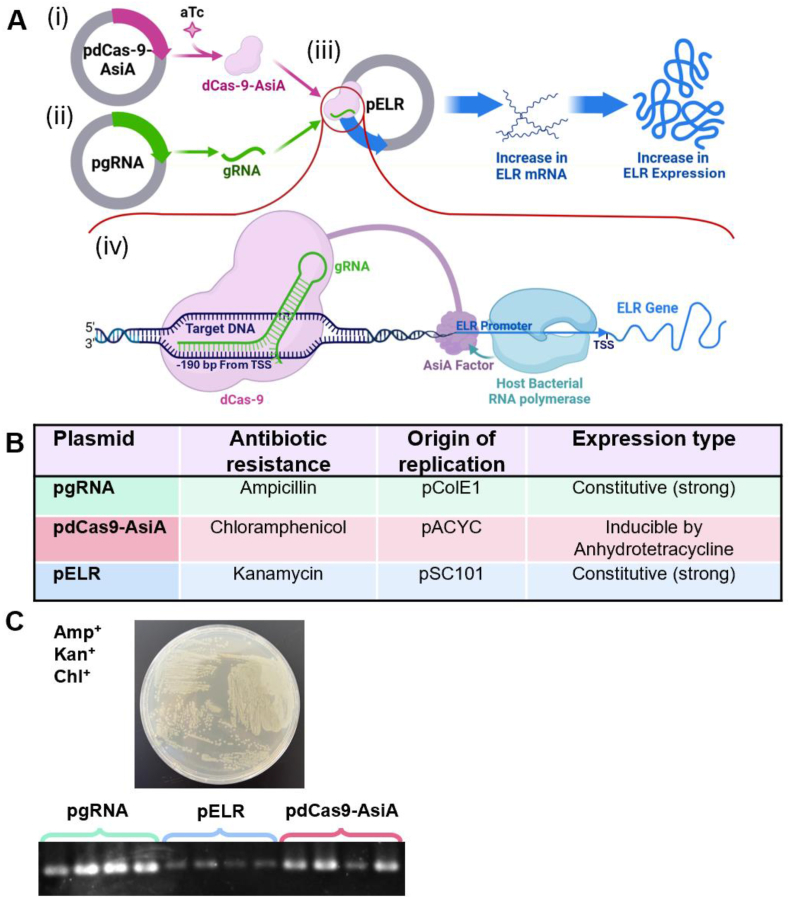
CRISPRa System in *E. coli*: Design, Stability, Functionality, and Validation. (A) Schematic representation of the three-plasmid CRISPRa system; (i) the pdCas9-AsiA plasmid, encoding a catalytically inactive Cas9 (dCas9), inducible by Anhydrotetracycline (aTc) fused to a mutant T4 phage anti-sigma factor AsiA-m2.1, which functions as a potent transcriptional activator; (ii) the pgRNA plasmid, encoding the guide RNA (gRNA) that targets a region 190 bp upstream of the ELR gene's transcription start site (TSS); (iii) the pELR plasmid, containing the target ELR gene; and (iv) The schematic representation of the mechanism of action of the CRISPRa system. Created with BioRender.com. (B) Regulatory elements for plasmid components of the CRISPRa system. (C) Verification of plasmid co-transformation and stability in *E. coli*. Clones were selected based on the simultaneous resistance to ampicillin, kanamycin, and chloramphenicol. Colony PCR was performed to confirm the presence of all three plasmids within the selected clones 48 h after the transformation.

To assemble all the components necessary for CRISPRa function, we developed a multi-plasmid system in which each plasmid carries a separate module of the system. Specifically, three plasmids were employed: one containing the Elastin-Like Recombinamer (ELR) gene (pELR), another encoding a catalytically inactive form of Cas9 (dCas9) fused to the mutant T4 phage anti-sigma factor AsiA-m2.1 (which acts as a strong transcriptional activator) (pdCas9-AsiA) [25], and a third encoding the guide RNA (pgRNA) [26]. The guide RNA was designed to target a specific sequence on the pELR plasmid, located approximately 190 bp upstream of the ELR transcription start site (TSS) (Fig. 1A).

To create a stable three-plasmid system, each plasmid was equipped with a unique antibiotic resistance gene and origin of replication, preventing replication incompatibility and supporting long-term plasmid stability. The expression of dCas9-AsiA was regulated by a PtetO induction system activated by anhydrotetracycline (aTc), while both the gRNA and the ELR gene were constitutively expressed from a strong promoter; BBa_J23119 and BBa_J23110 respectively (Fig. 1B).

These three plasmids were sequentially co-transformed into *Escherichia coli* BW25113. Bacterial clones were initially selected based on resistance to ampicillin, kanamycin, and chloramphenicol. To confirm the presence of all three plasmids, colony PCR was performed using primers designed to amplify a specific ∼500 bp fragment unique to each plasmid. Colony PCR of four antibiotic-resistant clones yielded positive amplification for all three plasmids in every tested clone 48 h after the transformation (Fig. 1C) and right after the liquid culture of the clones for ELR production (Fig. S1).

**Fig. 2:**
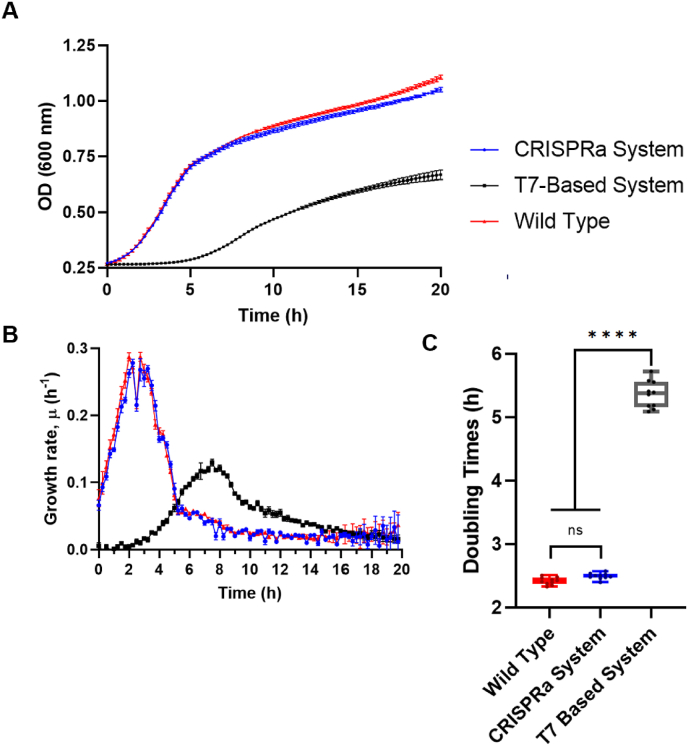
Growth kinetics comparison among CRISPRa, T7-based, and wild-type bacterial systems. (A) Optical density (OD600) measurements over time. (B) Growth rate over time analysis of the strains. Growth rate (μ) was calculated as the derivative of the natural logarithm of OD600 over time. (C) Doubling time of the strains during the exponential growth phase. Doubling time was determined as ln(2)/μ_max,_ where μ_max_ corresponds to the maximum growth rate during the exponential phase. Data represent mean ± standard deviation (n = 10). Statistical significance was determined using one-way ANOVA followed by Tukey's multiple comparison test, ∗∗∗∗p < 0.0001.

Additionally, we evaluated the growth kinetics of this triple-resistant strain in comparison with the wild type strain and a production strain (*E. coli* BLR) bearing the expression of the same ELR gene under the control of a T7 promoter, as one of the most traditional and popular expression systems. The results revealed that the strain bearing the CRISPRa system showed a typical bacterial growth curve similar to the one described by the wild type strain (Fig. 2A), indicating that the three-plasmid system did not impair bacterial fitness. This observation suggests that the plasmids remain compatible over time, maintaining resistance traits and stable coexistence within the host. In contrast, the T7-based system shows a marked growth delay and lower overall growth in absolute terms. This suggests that the activation of gene ELR gene expression through CRISPRa does not impose a significant metabolic burden on bacterial cells, whereas the T7-based system may exert a substantial fitness cost.

Growth rate analysis (Fig. 2B) further supports these observations. The CRISPRa system and the wild-type strain exhibit a rapid exponential growth phase, with peak growth rates reaching similar values. In contrast, the T7-based system demonstrates a delayed exponential phase and a lower maximum growth rate, indicating an overall reduction in cellular viability. This reduced growth rate is likely due to the transcriptional burden associated with the T7 promoter, which can lead to resource depletion and impaired cellular functions.

The doubling time analysis in the exponential growth phase (Fig. 2C) provides additional evidence of these growth disparities. The CRISPRa system and the wild type strain have statistically similar doubling times, confirming that CRISPRa activation does not negatively impact bacterial proliferation. However, the T7-based system exhibits a significantly prolonged doubling time, reinforcing the notion that T7-driven gene expression is associated with considerable growth retardation.

Collectively, these results confirm the successful co-transformation, compatibility, and stable presence of all three plasmids, underscoring the long-term stability of the CRISPRa system in the same bacterial cells. Additionally, CRISPRa system does not impose a significant metabolic burden and supports bacterial growth effectively. In contrast, the T7-based system exhibits clear signs of cellular toxicity and metabolic stress, leading to growth delays and reduced viability.

### 2.2 Enhanced ELR gene expression using the CRISPRa system

To investigate whether the CRISPRa system could enhance ELR gene expression, we compared a strain carrying only the plasmid encoding the ELR gene (pELR)—designated “No CRISPRa”—with a modified strain labeled “CRISPRa,” which also bears the pdCas9–AsiA and pgRNA plasmids designed to activate ELR transcription.

**Fig. 3:**
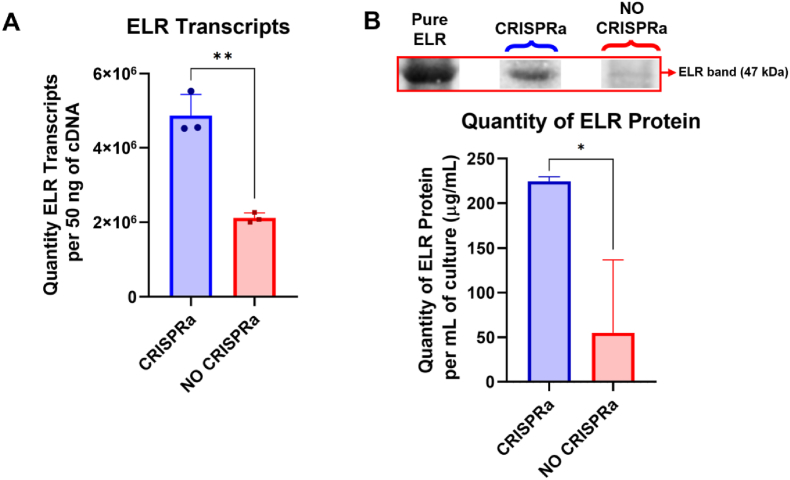
Analysis of ELR Gene Expression Enhanced by CRISPRa system at the Transcriptional and Protein Levels (A) Absolute qPCR analysis of ELR gene transcription in CRISPRa and No CRISPRa strains, copy number of transcripts for ELR gene per 50 ng of cDNA (n = 3). Statistical significance was determined using T-test, ∗∗p < 0.001. (B) SDS-PAGE analysis of protein lysates from CRISPRa and No CRISPRa strains, followed by quantification through densiometric image analysis (n = 3). The ELR band was identified based on a molecular weight comparison with pure ELR samples loaded on the same gel. The intensity of the ELR band in each sample was quantified using a standard curve of the pure ELR loaded in the same gel. Statistical significance was determined using T-test, ∗p < 0.01.

Absolute qPCR analysis of both strains revealed an approximately twofold increase in ELR transcript levels in the CRISPRa strain compared to the No CRISPRa control (Fig. 3A). These data indicate that the CRISPRa system effectively enhances ELR gene transcription.

To determine whether this transcriptional increase translates to higher protein levels, we performed SDS-PAGE on protein lysates from both strains (Fig. S2), followed by densitometric quantification. Consistent with the qPCR results, the CRISPRa strain exhibited about a 4-fold increase in ELR protein production, in comparison to the No CRISPRa control (Fig. 3B).

Collectively, these findings show that the CRISPRa system not only boosts ELR mRNA levels but also substantially improves the corresponding protein yields from approximately 50 mg–200 mg of ELR per L of culture. While the overall ELR production level may still be insufficient for establishing a fully optimized system, this proof of concept demonstrates the effectiveness of CRISPRa in increasing the recombinant production of ELRs.

## 3 Discussion

The demand for advanced biomaterials continues to rise across biomedical, pharmaceutical, and industrial sectors. Within this landscape, ELRs have garnered attention due to their unique mechanical and biological characteristics, which allow them to mimic natural tissue properties and offer tunable functionality. However, developing efficient expression systems for such complex recombinant proteins remains challenging. In this study, a CRISPRa system was employed to enhance ELR production at both the transcriptional and protein levels, showing an approximately twofold increase in ELR transcript abundance and a notable corresponding rise in ELR protein yield. In bacterial systems, a single mRNA molecule can be translated into multiple copies of the corresponding protein, meaning that even a modest increase in mRNA levels can lead to a relatively high increase in protein yield. This explains the observed discrepancy between the twofold increase in mRNA transcription and the 4-fold increase in protein production, which is consistent with the expected dynamics in bacterial protein expression systems.

These findings emphasize the promise of CRISPRa as a modular tool for boosting the production of protein-based biomaterials. By taking advantage of a three-plasmid co-transformed bacterial system, it was possible to achieve targeted transcriptional activation of the ELR gene, illustrating the feasibility of this approach for applications requiring moderate yet specific upregulation of recombinant protein expression. From a broader perspective, employing CRISPRa in bacterial hosts may open opportunities for scaling up biomaterial production to meet industrial demands.

Nevertheless, several limitations must be considered before this system can be adopted more broadly. First, although CRISPRa improved ELR yields, the absolute protein quantity achieved in this study remains below what would be necessary for a fully optimized production process. Second, the multi-plasmid strategy, while effective here, may pose challenges related to plasmid compatibility and metabolic burden under large-scale fermentation conditions. The dependence on specific host strains also introduces variability, suggesting a need to test alternative strains or engineering approaches to enhance robustness and consistency.

To advance this technology toward industrial application, further refinements should focus on improving yield, stabilizing plasmids, and reducing proteolytic degradation. Tailoring guide RNA design, optimizing the distance between the activator complex and the transcription start site, and exploring stronger or more finely tuned promoters may significantly bolster expression. Concurrently, evaluating different fermentation strategies—such as using specialized media or adopting engineered host strains with reduced protease activity—may further enhance productivity. Employing computational modeling and high-throughput synthetic biology approaches could also accelerate iterative improvements.

Taken together, these considerations highlight the potential for CRISPRa to become a powerful platform for the production of ELRs and other protein-based biomaterials, offering a level of transcriptional control that can be adapted to diverse applications. Future efforts directed at overcoming the outlined limitations will help transform this proof of concept into a robust and scalable system, ultimately broadening the availability of next-generation biomaterials.

## 4 Materials and methods

### 4.1 *E. coli* strains and culturing conditions

All plasmids were maintained and constructed separately in XL1-Blue *E. coli* Cloning-Grade Competent Cells (Agilent technologies) prior usage in experimental strain. During plasmid design process, bacteria was grown in LB medium (Luria-Bertani Media) at 30 °C with agitation 250 rpm. Experiments were conducted in BW25113 strain (*E. coli*), which was derived from *E. coli* K-12, specifically from MG1655 through modification in metabolic operons (araBAD, rhaBAD) and ribonuclease (rph) gene. For assessing protein recombinant production bacterial cells were grown in 10 ml of TB (Terrific Broth Media) medium at 30 °C with agitation 250 rpm, this temperature is chosen because the SC101 origin of replication, which is part of the CRISPRa plasmid system, is more active at this specific temperature [27], leading to more plasmid stability. Both strains were chemically competent for introducing plasmids containing components of the CRISPR activation (CRISPRa) system using standard cloning methods. Plasmids were maintained under antibiotic selection with antibiotic concentrations: chloramphenicol (Chl) 25 μg/ml, kanamycin (Kan) 50 μg/ml, and ampicillin (Amp) 100 μg/ml. Induction of the CRISPRa system, specifically the induction of the transcription of dCas-9-AsiA gene, was achieved by adding anhydrotetracycline (aTc) at a concentration of 100 ng/ml to the bacterial cultures during the exponential growth phase, following 10 h of culturing. After 5 h of growth with aTc, the bacteria were harvested for further characterization.

Additionally, for the comparison with the traditional recombinant expression system, the ELR gene was introduced into a modified pET-25(+) expression vector and then transformed into the *E. coli* strain BLR(DE3)star (Invitrogen). This strain was grown under the same conditions as previously described for ampicillin selection and culture medium.

### 4.2 Plasmids for the CRISPRa system

For the experiments conducted in BW25113 (*E. coli*), three distinct plasmids were employed. The first plasmid, derived from the pColE1 origin of replication, presents a moderate-to-high copy number and encoded the gRNA190, which enabled dCas9-AsiA-m2.1 to bind 190 bp upstream of the transcription start site (TSS) on the plasmid containing the target gene. This plasmid, which also conferred ampicillin resistance, was derived from the pgRNA-bacteria plasmid (a gift from Stanley Qi; Addgene plasmid #44251; http://n2t.net/addgene:44251; RRID:Addgene_44251) by using standard molecular biology and genetic engineering technics. The second plasmid was based on the pACYC origin of replication, exhibiting a moderate-to-low copy number. It encoded the dCas9-AsiA-m2.1 protein under the control of an aTc-inducible promoter and conferred chloramphenicol resistance. This plasmid, known as pdCas9- AsiA_m2.1 was a gift from Harris Wang (Addgene plasmid # 158065; http://n2t.net/addgene:158065; RRID:Addgene_158065). The third plasmid called pSKJ-E50I60 utilized the pSC101 origin of replication, which supports a low copy number, and encoded the target gene, E_50_I_60_, a member of the elastin-like recombinamers (ELRs) protein polymer family, while conferring kanamycin resistance. This plasmid was derived from commercial pET25(+) (Novagen) vector by changing the origin of replication, antibiotic resistance and promoter, moreover, ELR gene was inserted in the MCS, these modifications of the vector backbone were achieved by using standard molecular biology and genetic engineering technics. Importantly, the origins of replication of the pgRNA, pdCas9-AsiA-m2.1, and pSKJ-E50I60 plasmids belonged to different compatibility groups, ensuring stable maintenance and functional expression of all three plasmids within a single bacterial cell.

### 4.3 Evaluation of the three-plasmid system stability

To assess the stability of the CRISPRa system for inducing transcription of E_50_I_60_, firstly, sequential transformation of *E. coli* BW25113 with 3 plasmids: pgRNA-190, pdCas9-AsiA-m2.1, pSKJ-E50I60 was performed. 4 colonies resistant to the 3 antibiotics (Amp, Kan and Chl) were selected and then colony PCR was assessed to verify the presence of the 3 plasmids in each of the colonies 48 h after the transformation and right after the liquid culture of the clones for the ELR production. Three primer sets, which are listed in Table 1, were designed to amplify region targeting each of the three plasmids and colony PCR was performed, using DreamTaq Green PCR Master Mix (2X), normal thermal cycling parameters and an annealing temperature of 55 °C.

**Table 1:** Oligonucleotide primers used for Colony PCR Verification of the Three-Plasmid CRISPRa System in *E. coli* BW25113.

Primers	Sequence of Nucleotides (nt)	PCR Product length (bp)
pdCas9-AsiA-m2.1 forward	5′-AGTCTGAAGAAACAATTACCCC -3′	505
pdCas9-AsiA-m2.1 reverse	5′-AGTCTTTCCTCAATCATCTCCC -3′	
pgRNA-190 forward	5′- CGAGCATCACAAAAATCGAC -3′	528
pgRNA-190 reverse	5′- CAAACAAAAAAACCACCGCTAC -3′	
pSKJ-E50I60 forward	5′- CGAAACACGGAAACCGAAG -3′	514
pSKJ-E50I60 reverse	5′- GCGCAACGCAATTAATGTAAG -3′	

### 4.4 Evaluation of bacteria growth rate

The *E. coli* BW25113 strain, transformed with three plasmids (CRISPRa strain), *E. coli* BW25113 wild type strain and the *E. coli* strain BLR(DE3)star (Invitrogen), transformed with a modified pET-25(+) expression vector expressing the ELR protein under the control of a T7 promoter were cultured under experimental conditions (TB medium, 30 °C, agitation at 250 rpm in 96 well plates with volumes of 0.2 ml). Optical density (OD) was measured every 15 min (Wavelength 600 nm SpectraMax iD3). The exponential phase was identified for defining the estimated proper timing for adding aTc as the inductor of the CRISPRa system.

### 4.5 Quantification of gene expression induced by CRISPRa

Cell cultures were harvested after 5 h of induction with aTc. Afterward, total RNA was extracted using an Aurum Total RNA Mini Kit (Bio-Rad). 1 μg of RNA was converted into cDNA with a cDNA Synthesis Kit (Bio-Rad). qPCR was performed using TaqMan Universal Master Mix II, Taq-probe in 20 μL reaction volume and 60 °C annealing temperature. qPCR reactions were performed in triplicates on a 7500 Fast Real-Time PCR System using 50 ng of cDNA, 900 nM primer concentration y 250 nM Taq-probe concentration (Table 2). The expression levels of each gene were calculated based on a standard curve prepared using known concentrations of a PCR product containing the gene of interest. Control samples without a template were used to control gene-specific amplification (see Table 3).

**Table 2:** Oligonucleotide primers and Taq-probe used for qPCR.

Primers	Sequence of Nucleotides (nt)	PCR Product length (bp)
qPCR forward	5′-GATGTCGAGCACCACCAC-3′	70
qPCR reverse	5′-CCAACTCAGCTTCCTTTCGG-3′	
Taq-probe	5′-ACCACCACTGAGATCCGGCTGC-3′

**Table 3:** Oligonucleotide primers used to generate cDNA template for standard curve.

Primers	Sequence of Nucleotides (nt)	PCR Product length (bp)
Standard curve forward	5′-CTGGTGTATGAGTATGATCCG-3′	203
Standard curve reverse	5′-GCTAGTTATTGCTCAGCGG-3′	

### 4.6 Evaluation of protein expression induced by CRISPRa

To compare the yield of ELR (E_50_I_60_) in a bacterial expression system with and without the CRISPRa system, SDS-PAGE was performed using a 12 % polyacrylamide gel and cooper staining. Protein expression levels were quantified by analysing SDS-PAGE images using Image Lab 6.0 software. A standard curve for ELR was first generated by correlating the protein concentration (μg/mL) with band intensity on the gel images. This standard curve was then used to determine the concentrations of E_50_I_60_ in both expression conditions, enabling a quantitative comparison of protein yield.

## CRediT authorship contribution statement

**Pablo Rodríguez-Alonso:** Writing – review & editing, Writing – original draft, Visualization, Methodology, Investigation, Conceptualization. **Viktoriya Chaskovska:** Writing – original draft, Visualization, Methodology, Investigation. **Desiré Venegas-Bustos:** Writing – review & editing, Methodology, Investigation. **Alba Herraiz:** Writing – review & editing, Supervision, Methodology. **Matilde Alonso:** Writing – review & editing, Supervision, Methodology, Funding acquisition. **Jose Carlos Rodríguez-Cabello:** Writing – review & editing, Supervision, Project administration, Methodology, Funding acquisition, Conceptualization.

## Declaration of competing interest

The authors declare the following financial interests/personal relationships which may be considered as potential competing interests: Jose Carlos Rodríguez Cabello reports financial support was provided by 10.13039/501100007515University of Valladolid. Matilde Alonso Rodrigo reports financial support was provided by 10.13039/501100007515University of Valladolid. If there are other authors, they declare that they have no known competing financial interests or personal relationships that could have appeared to influence the work reported in this paper.

## Data Availability

Data will be made available on request.
