# Supplementary material for: Assessing the feasibility of CRISPRa approaches to enhance protein-based biomaterial expression in bacterial systems for more efficient production
Source: Mater Today Bio. 2025 Mar 29;32:101720. doi: 10.1016/j.mtbio.2025.101720 (PMC11997408; doi:10.1016/j.mtbio.2025.101720)
Supplement: Multimedia component 1 [file mmc1.pdf]

Supplementary Figures For

Assessing the Feasibility of CRISPRa Approaches to Enhance Protein-Based Biomaterial Expression in Bacterial Systems for More Efficient Production.

Authors

Pablo Rodríguez-Alonso,[1,2]† Viktoriya Chaskovska,[1]† Desiré Venegas-Bustos,[1] Alba Herraiz,[1] Matilde Alonso,[1] Jose Carlos Rodríguez-Cabello[1]*

† These authors contributed equally to this work
* Correspondence: Jose Carlos Rodríguez-Cabello (carlos.roca@uva.es)

Affiliations
[1] Bioforge Lab (Group for Advanced Materials and Nanobiotechnology), Laboratory for Disruptive Interdisciplinary Science (LaDIS), CIBER-BBN, Edificio LUCIA, Universidad de Valladolid, Valladolid, 47011, Spain.
[2] Technical Proteins Nanobiotechnology S.L., Valladolid, Spain.

The PDF file includes:

Fig. S1. Colony PCRs of triple resistant clones.
Fig. S2. SDS-PAGE of bacterial lysates.

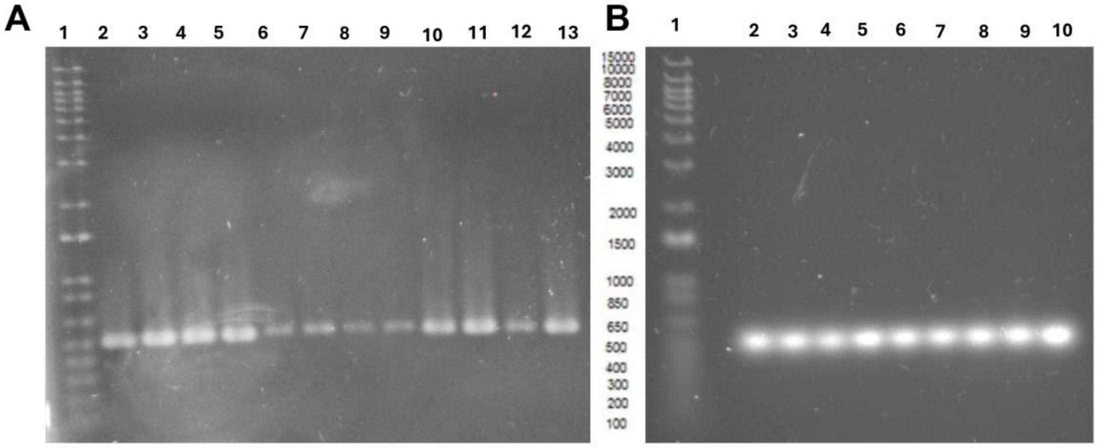

Fig. S1. Colony PCRs of triple resistant clones. (A) 48 h after the transformation. 1. Marker; 2-5. pgRNA; 6-9. pELR; 10-13. pdCas9-AsiA. (B) Right after the liquid culture of the clones in for ELR production. 1. Marker; 2-4. pgRNA; 5-7. pELR; 8-10. pdCas9-AsiA. Molecular ladder for DNA fragment size is provided in the middle of the figures (BPs)

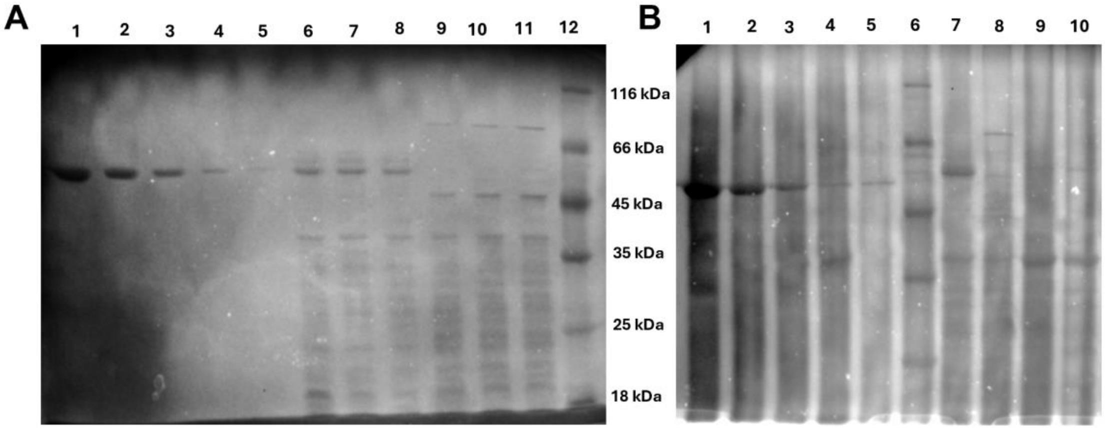

Fig. S2. SDS-PAGE of bacterial lysates. (A) 1-5. $E_{50}I_{60}$ curve for quantification (500, 250, 125, 50, 25 µg/ml); 6-8. CRISPRa system replicates; 9-11. Wild type strain; 12. Protein Marker. **(**B) 1-5. $E_{50}I_{60}$ curve for quantification (500, 250, 125, 50, 25 µg/ml); 6. Protein Marker; 7. Wild type strain; 8-10. No CRISPRa system replicates. Molecular ladder for protein size is provided in the middle of the figures (kDa).
